# Population Health Rankings as Policy Indicators and Performance Measures

**Published:** 2010-08-15

**Authors:** Thomas R. Oliver

**Affiliations:** University of Wisconsin School of Medicine and Public Health, University of Wisconsin Population Health Institute

## Abstract

Population health rankings can be used by various actors for different purposes. This article examines those potential uses and concludes that the chief promise of population health rankings lies in 2 areas. The first is to help set agendas — stimulating awareness, motivation, and debate over means to improved health outcomes. The second is to help establish broad responsibility for population health and the need for multisectoral collaboration to improve outcomes. A new performance regime based on rankings will require more research to establish causal pathways and relative determinants of health, as well as stronger evidence about the effects of public and private interventions to guide investment strategies. Finally, leaders who develop and promote population health rankings must further develop the technical community needed to translate the response to the rankings into constructive public debate and policy development.

## Introduction

Citizens and their leaders are bombarded by information about individual and population health. Information comes via scientific reports, mass media, commercial advertising, government surveys and statistics, and simple word of mouth from family, friends, colleagues, and acquaintances. What information do they pay attention to and with what consequences?

The question is relevant because population health improvement relies on the skillful production and effective use of information. A core element of a new initiative, Mobilizing Action Toward Community Health (MATCH), involves the development and dissemination of population health rankings for all counties in the United States. These rankings are one of several recent efforts to systematically measure and compare the population health or health systems of countries, states, and communities. They represent one category of research-related activities needed to better establish relationships among the multiple determinants of health and guide future investment for population health improvement (1).

This article examines how population health rankings can inform and structure public debate and policy development. It considers how such rankings are a form of *policy indicators*, measures that help monitor social conditions and at times prompt action to improve those conditions (2). It also considers such rankings as part of a *performance regime* intended to draw greater attention to, and establish greater accountability for, population-wide health outcomes (3,4). In these roles, rankings serve primarily as a tool for democratic governance of a complex system that affects population health rather than management of a specific public health agency or program (5,6).

## Possible Uses for and Responses to Population Health Rankings

Population health rankings combine both forms of what Charles Lindblom and David Cohen referred to as "professional social inquiry": 1) systematic data gathering and reporting and 2) statistical manipulation and analysis of social data (7). Although there are modest methodologic differences, the new MATCH rankings are modeled after the county health rankings developed by the University of Wisconsin Population Health Institute (UWPHI) (8).

The MATCH project, a collaboration of UWPHI and the Robert Wood Johnson Foundation, has produced 50 state reports, ranking counties from first to last in each state (9). The rankings are created on the basis of current *health outcomes* (5 measures of premature death, self-reported health, and birth outcomes) and *health factors* as predictors of future health outcomes (23 measures related to clinical care [20% of total], health behaviors [30% of total], social and economic factors [40% of total], and physical environment [10% of total]). Each county receives an overall ranking for health outcomes and health factors, as well as a rank for each category of health factors (9).

Overall, the rankings "are designed to summarize the current health of the counties, as well as the distribution of key factors that determine future health" (8). More specifically, they are intended to raise "awareness of variation in populations' health [and] appreciation of the variety of factors that affect populations' health and that are amenable to influence by public- and private-sector programs and policies" (10). As such, they are a potentially important contribution by researchers, serving what Carol Weiss calls the "enlightenment function" in shaping public understanding and policy debates (11,12).

However, the influence of population health rankings is potentially much broader and more complex. Population health rankings or other system performance measures are more than expert analysis of social conditions; they constitute a political act. Deborah Stone explains, "Measures imply a need for action, because we do not measure things except when we want to change them or change our behavior in response to them." Moreover, "Counting is often part of a deliberate effort to stimulate creation of a natural community by identifying a statistical community in order to demonstrate common interests" (13). Indeed, the UWPHI intends that its county health rankings help establish stronger community identity and collaboration: "By taking a broad perspective on the factors that influence health — health care, health behaviors, socioeconomic factors, and the physical environment — we hope to encourage all community stakeholders to work with health departments and health care providers as partners in the public health system." (8).

The key message of this article is that a set of public statistics, whether produced by public or private organizations, can be used by various actors for different purposes. The data can be used by individuals and organizations acting alone or participating in a collective decision. Those actors, in turn, can be motivated by self-interest or their vision of the general welfare.

### Multiple audiences for performance measures

Most performance measures, whether in public health or another social domain, have many potential target audiences (5), 3 of which are the following:

1. The general public, as citizens or consumers of services. The mass media play a role in mediating between the production of performance measures and the public response to them.
2. The community of experts, including scientists, policy analysts, service providers, and other stakeholder groups and organizations. These actors are critical in legitimizing perceptions of a problem and refining the problem definition, as well as responding to a perceived problem with plausible solutions.
3. Policy makers in both public and private institutions whose priorities and leadership skills shape the nature and degree of response to performance measures, including shifting resources, altering incentives, or avoiding blame. Policy makers at different levels — local, state, or national — have different responsibilities and different policy tools at their disposal.

The level of awareness, likelihood of response, and capacity for effective response vary depending on the audience. Furthermore, a given audience is likely to be more influential in certain aspects of the policy process than in others.

### Multiple uses for performance measures

Technical information and analysis, however skillfully prepared and relevant, usually play a limited role in social problem solving (7). Decision makers routinely arrive at decisions by relying on ordinary knowledge and learning through experience and through interactions that are driven by political compromise. Nonetheless, scholars have identified many ways in which performance measures shape the process of policy development.

**Problem identification and agenda setting**

Population health rankings, as policy indicators, help to identify and define problems and thereby help set the policy agenda. Indicators can serve as a "warning" to policy makers and move a particular issue higher on their list of priorities (5,11,14,15). They provide an opportunity for media attention and advocacy to spotlight a problem, frame it, and create new venues for action (16,17).

The creators of the Wisconsin County Health Rankings suggest that the simplicity and competitive nature of those performance measures have translated into considerable media attention across the state. That attention has been parlayed by county health officers into many uses, the most frequent being educating county board members or other policy makers in their community (18).

The county rankings, both in Wisconsin and now in MATCH, are intended to be produced and disseminated annually. The longitudinal nature of the enterprise raises the question of what will have the most impact: the initial county health rankings or subsequent changes in counties' rankings over time. The impact may depend on the stability of the rankings' methods and its inputs for health factors and health outcomes. Changes in the composite measures must be shown to reflect genuine changes in performance rather than random statistical variation (6,19). If counties' rankings are highly volatile from year to year, their validity and importance may be discounted. If instead they are seemingly immutable over several years despite what local officials consider to be concerted efforts to improve population health, then they may also come to be distrusted and discounted. Another factor in the influence of the rankings may be how familiar local policy makers are with them and whether the creators of the rankings or other intermediaries have been able to engage and educate receptive experts and community leaders on their value (10,18).

**Policy design**

A second use of population health rankings or other performance measures is providing what Weiss calls "guidance" to policy makers in how to respond to widely acknowledged problems (11,12). The critical issue here is not responsiveness but effectiveness; if leaders do something when confronted with a serious social condition, will their response solve or at least reduce the extent of the problem? This role depends heavily on experts, on whom leaders rely to develop and evaluate options for new policies and programs (14,20).

Donald Moynihan also describes how performance information (eg, rankings, report cards) can have a "purposeful" use in improving existing programs and service delivery. "Performance data would be used to better allocate resources, make decisions about strategy, reengineer processes, motivate workers, and usher in a new era of accountability" (4).

The capacity of population health rankings to guide policy design depends on the awareness users have of the underlying model of health determinants. In the UWPHI model, separate measures of socioeconomic conditions, behaviors, environmental conditions, and health services alert users to which of those factors boost or weaken the overall county ranking. An accompanying database describes an array of policies and programs and the strength of the evidence of their potential effect on health outcomes (21). What is not yet established is whether community or state leaders believe they have necessary guidance from the rankings and menu of potential interventions to invest in options most likely to improve population health or, even if they have necessary information, whether it is insufficient because other obstacles prevent their communities from adopting the most effective options for population health improvement. As noted earlier, policy makers and organizational leaders will pursue changes even without solid knowledge of the causal factors that underlie the performance measures (3,7).

**Policy adoption**

A third use of the information provided by population health rankings is overtly political: contesting parties may use the rankings as "ammunition" to support their established policy and programmatic preferences (3,4,11). One would expect that groups or organizations that are either beneficiaries of programs related to population health or potential targets of regulation would be most likely to mobilize in response to performance measures and attempt to use them to their advantage. There is little empiric evidence about whether stakeholders will mobilize and whether this mobilization is focused on narrow problems and populations or broader-based collaboration and action.

## Usefulness and Effectiveness of Performance Measures

Organizations that are responsible for a community's health are perhaps the most important users of population health rankings or related performance measures. How county boards, public health departments, and health care organizations respond to rankings is likely to critically affect public debate and policy development.

Responses can be active or passive, functional or dysfunctional. Functional responses focus on process improvement, input reallocation, management focus and style, and mission enhancement. Dysfunctional responses focus on "cream-skimming," deception, and blaming the messenger for poor performance (4,5,22). Organizations may resort to goal displacement, shifting their focus to outcomes with more favorable performance measures or over which they have more control (3). These dysfunctional responses are most likely to occur when measures are linked with substantial incentives, are easy to manipulate, or omit important factors in performance.

### Communicating with target audiences

The responses of community leaders and other stakeholders are heavily influenced by the quality of the performance information and the context in which it is used. In their detailed study of organizational report cards, Gormley and Weimer found that effectiveness was related to the content (validity and comprehensiveness), communication (comprehensibility and relevance to the appropriate audience), and capacity for organizational responses (reasonableness and functionality) (5).

The developers of the UWPHI population health rankings have emphasized comprehensiveness and comprehensibility. In its current state, the science is more vulnerable to attacks on its credibility. Stone warns, "Numbers can create the illusion that a very complex and ambiguous phenomenon is simple, countable, and precisely defined" (13). Similarly, Lindblom and Cohen urge researchers to avoid a "misplaced pursuit of authoritativeness"; they believe technical analysis is most influential when it confirms the ordinary knowledge of citizens, policy makers, and other issue experts (7). The UWPHI developers are careful to acknowledge the methodologic limitations and suggest the rankings are just one of many tools that should be used for community health assessment and improvement (18).

Perhaps the biggest challenge is communicating the relevance of population health rankings to leaders and organizations outside the conventional boundaries of public health and encouraging multisectoral responsibility for health determinants and outcomes (23). Complex systems and programs limit accountability and make the use of performance measures more difficult because verifying whether and how inputs and outputs connect to outcomes is difficult (4,24,25). Conversely, developing measures of system performance that connect a range of inputs, such as education, housing, and environmental conditions, to population health may attract attention from a wider set of organizations and leaders. In fact, extending outcome measures beyond population health to more general values, such as net economic benefit, subjective well-being, and equity, is desirable (2).

### Causal models to guide action

To guide both expert analysis and public debate through the challenges posed by complex systems, it is necessary to pair policy indicators, such as population health rankings, with causal models. "[Policy choice] requires not only information on conditions of well-being or justice but also causal knowledge about how to promote them" (2).

Strengthening the underlying causal model of health determinants is critical to the long-term influence of population health rankings because the contributions of each major category of determinants and the weight of measures in each category require further empirical validation (1). Furthermore, attention must be paid to the sensitivity of composite measures to changes in the weighting structure (19). Incomplete measurement, uncertain weighting of measures, and distance of measured inputs from desired outcomes are common problems in systems of performance measurement (5).

### Incentives for improving performance

A final concern is whether the incentives established by performance measures are properly aligned with the goal of population health improvement. Information about organizational performance, or community performance, must affect the flow of resources through either consumer decisions or public budgets (5).

One problem is that community health has little competition for customers. Advocates for population health rankings cannot hope to achieve the same response as, say, standard achievement scores for public school districts and individual schools. However, population health rankings conceivably may affect prospects for local economic development by influencing recruitment and retention of employers.

Another problem is that community leaders or public health officials who face evidence of poor performance may argue that rankings or scorecards actually measure community needs, not system performance. Measures are often used to legitimize or dispute claims for resources and privileges (13). Communities with effective advocates or strong stakeholders may secure more resources, and those that lack strong leadership and stakeholders will likely suffer a further loss of resources and have continued poor performance.

Performance assessment can have some positive effect if the results threaten the reputation of organizational and community leaders. Fear of embarrassment is perhaps the most powerful motivator for organizational leaders. Reputation affects leaders' professional standing among peers, organizational morale, degree of oversight from government officials and consumer advocates, and managerial discretion for leaders over resources and operations (5,26-29).

Although powerful incentives for population health improvement may be desirable, they carry the risk of being manipulated either to protect personal reputations or promote favored courses of action (22). Duncan MacRae, Jr, stresses the importance of selecting indicators that are not susceptible to bias from entities that produce data to be used in performance measurement or that have the potential to be affected by the performance measures (2). To avoid efforts to alter or obscure population health rankings, researchers should engage community leaders and especially their public health colleagues to convert attention from poor performance into a renewed commitment for collaboration and improvement. An interactive dialogue among key stakeholders has the potential to foster both shared mental models and stronger commitment to performance (3).

## Implementation of Population Health Performance Measures

MacRae argues that researchers and others interested in establishing new indicator systems must recognize certain key features of the process. First, the process is both political and technical; indicators cannot be developed or sustained without input from interested groups as well as experts. Second, institutionalizing an indicator at the national level may take a long time, even decades. Trial and experimentation are essential for testing usefulness, and local experience can facilitate later national use. Third, indicators may prove to be less intelligible and less relevant to policy than initially thought, and widespread adoption and use are likely to require advance testing of reliability and relevance. Finally, different political communities — particularly states and localities — have different goals and means of action and, therefore, different information needs. For all these reasons, he argues that we "must be skeptical of rapid development of practical information systems, and of design by experts without continuing participation of users" (2).

## Conclusion

Rankings can serve an important function in the development of a new performance regime that is dedicated to population health improvement. Population health rankings contribute to agenda setting — stimulating awareness, motivation, and debate over means to improved health outcomes — and help establish broad responsibility for population health and the need for multisectoral collaboration to improve outcomes and future rankings. However, the comprehensiveness of the new paradigm is a double-edged sword. Rankings and other population performance measures are limited by the complexity and ambiguity of accompanying models of health determinants, which in turn may limit broad mobilization, selection of policy options, and clear accountability for results. Therefore, a new performance regime will require further research to establish causal pathways and determinants of health, as well as stronger evidence on the effect of public and private interventions to guide investment strategies.

Expecting the general public to be the impetus for population health improvement is unrealistic. Action and long-term improvement depend heavily on the relationships between health leaders and other community leaders and those between state and local leaders. Improvement also depends on incentives, material and nonmaterial, to address threats to health outcomes and monitor trends. The most powerful motivation for improvement may be the reputations shared by state and local leaders based on the publicity associated with population health rankings.

Finally, public health leaders who develop and promote population health rankings must also expand and strengthen the technical community that is needed to translate the response to the rankings into constructive public debate and policy development. Communication with the public, mass media, and political leaders outside public health is critical for establishing a new paradigm of thought and action that recognizes health as more than health care and infectious disease control.
